# Guidelines on Chemotherapy in Advanced Stage Gynecological Malignancies: An Evaluation of 224 Professional Societies and Organizations

**DOI:** 10.1371/journal.pone.0020106

**Published:** 2011-05-17

**Authors:** Nikolaos P. Polyzos, Davide Mauri, John P. A. Ioannidis

**Affiliations:** 1 Department of Hygiene and Epidemiology, University of Ioannina School of Medicine, Ioannina, Greece; 2 Department of Medical Oncology, General Hospital of Lamia, Lamia, Greece; 3 Stanford Prevention Research Center, Stanford University School of Medicine, Stanford, California, United States of America; 4 Department of Medicine, Tufts Medical Center, Institute for Clinical Research and Health Policy Studies, Tufts University School of Medicine, Boston, Massachusetts, United States of America; Copenhagen University Hospital, Denmark

## Abstract

**Background:**

Clinical practice guidelines are important for guiding practice, but it is unclear if they are commensurate with the available evidence.

**Methods:**

We examined guidelines produced by cancer and gynecological societies and organizations and evaluated their coverage of and stance towards chemotherapy for advanced stage disease among 4 gynecological malignancies (breast, ovarian, cervical, endometrial cancer) where the evidence for the use of chemotherapy is very different (substantial and conclusive for breast and ovarian cancer, limited and suggesting no major benefit for cervical and endometrial cancer). Eligible societies and organizations were identified through systematic internet searches (last update June 2009). Pertinent websites were scrutinized for presence of clinical practice guidelines, and relative guidelines were analyzed.

**Results:**

Among 224 identified eligible societies and organizations, 69 (31%) provided any sort of guidelines, while recommendations for chemotherapy on advanced stage gynecological malignancies were available in 20 of them. Only 14 had developed their own guideline, and only 5 had developed guidelines for all 4 malignancies. Use of levels of evidence and grades of recommendations, and aspects of the production, implementation, and timeliness of the guidelines did not differ significantly across malignancies. Guidelines on breast and ovarian cancer utilized significantly more randomized trials and meta-analyses. Guidelines differed across malignancies on their coverage of disease-free survival (p = 0.033), response rates (p = 0.024), symptoms relief (p = 0.005), quality of life (p = 0.001) and toxicity (p = 0.039), with breast and ovarian cancer guidelines typically covering more frequently these outcomes. All guidelines explicitly or implicitly endorsed the use of chemotherapy.

**Conclusions:**

Clinical practice guidelines are provided by the minority of professional societies and organizations. Available guidelines tend to recommend chemotherapy even for diseases where the effect of chemotherapy is controversial and recommendations are based on scant evidence.

## Introduction

Clinical practice guidelines are important for interpreting and translating research evidence for clinical practice and medical decision making. As stated by the Institute of Medicine “guidelines are systematically defined and evidence-based statements that reduce undesirable practice variation by discouraging use of services of questionable value while encouraging use of services with proven efficacy” [1]. However developing reliable, updated and uniform guidelines requires great care and commitment and professional organizations often do not offer detailed recommendations for certain diseases or groups of patients [2]. Moreover, even when guidelines are available, it may be difficult to locate guidelines developed by diverse organizations [3]. For some diseases and questions of interest, sufficient and/or good quality scientific evidence about what to recommend is lacking [4] and subjective recommendations may sometimes be inconsistent across guidelines by different societies, or even harmful [5], [6]. Even when extensive, high-quality evidence exists, there is no assurance that different guidelines make similar recommendations [7] and timely updates are also essential [8], [9], especially when evidence is changing rapidly. When there are guidelines on the same question of interest produced by different organizations, discrepancies in recommendations may generate confusion.

Here we aimed to examine systematically guidelines produced by professional societies and organizations in the fields of cancer and gynecological oncology and evaluate in depth guidelines that address the use of chemotherapy in advanced stage gynecological malignancies, including breast, ovarian, cervical and endometrial cancer. All these malignancies are common [10]. Importantly, they respond differently to chemotherapy, with extensive evidence and clear benefits demonstrated for breast and ovarian cancer and questionable benefits, for advanced endometrial and cervical cancer. Moreover, these malignancies differ a lot in the number of randomized trials that have been conducted on chemotherapy: recent meta-analyses have identified 370, 198, 65, and 13 randomized trials on chemotherapy for advanced stage breast, ovarian, cervical, and endometrial cancer, respectively [11], [12], [13], [14] and the evidence suggests that chemotherapy can be useful for advanced breast and ovarian cancer, but net benefits for hard endpoints (e.g. survival) are limited, if any, for stage III–IVA cervical cancer, and probably trivial or non-existent for stage IVB cervical cancer and advanced endometrial cancer [11], [12], [13], [14]. We performed an extensive search to catalogue relevant societies and organizations worldwide and examined which of them provide any sort of clinical practice guidelines pertinent to chemotherapy for these malignancies. These guidelines were then scrutinized to assess and compare their features and concordance with the available evidence.

## Methods

### Identification of pertinent societies and organizations

We constructed a database of gynecological and cancer societies and organizations that might provide guidelines for chemotherapy in advanced gynecological malignancies. We considered societies and organizations that were international (with global outlook), intercontinental (including two or more countries in a continent), or national belonging to one of the top 20 countries with the highest human development index [15]. We did not consider private institutions, regional or local societies, regardless of whether they might have produced guidelines or not. Organizations could be either professional societies for specialists or other public not-for-profit organizations.

We performed internet searches in Google and Yahoo (completed in June 2009). Initially we made 624 different searches with each engine and the first 100 results for each search were scrutinized. The 624 searches pertain to all the possible combinations of 8 subject matter terms (“cancer”, “oncology”, “medical oncology”, “radiation oncology”, “surgical oncology”, “cancer research”, “gynecology”, “gynecologic oncology”), three terms for type of entity (“society”, “association”, “organization”), and 26 terms for geographic identifiers (“International”, “European”, “Asian”, “African”, “American”, “Australasian and “Oceanian” and other eligible countries' names). Whenever we came across a URL referring to a potentially pertinent society or organization, we searched the entire directory. All the links in these websites were further searched in order to reach any additional pertinent societies or organizations. We recorded both organizations with accessible webpages, as well as those whose presence was mentioned in some URL, but either their link was not functional (not working or under construction) or they did not have a webpage that we could identify.

### Identification of guidelines from pertinent organizations

Available websites from eligible societies and organizations were scrutinized for any sort of clinical practice guidelines on any subject matter (last update June 2009). Whenever there was availability to perform an electronic search within the website, we used the terms “guidelines” or “recommendations” or “position statements” in English. For non-English websites, we translated these terms into the language the website used. We could do this in all languages except for 4 Japanese, 1 Swedish, 1 Finnish, 1 Danish and 2 Dutch organizations.

Additionally, we perused some standard websites containing information and/or links to guideline-related information, including the National Guidelines Clearinghouse (NGC) (www.guideline.gov), Guidelines International Network (G-I-N) (www.g-i-n.net), National Institute for Health and Clinical Excellence (NICE) (www.nice.org.uk), National Library of Health (www.library.nhs.uk), New Zealand Guidelines Group (NZGG) (www.nzgg.org.nz), Guidelines Advisory Committee (GAC) (www.gacguidelines.ca), Australian National Health and Medical Research Council (NHMRC) (www.nhmrc.gov.au) and the Scottish Intercollegiate Guidelines Network (SIGN) (www.sign.ac.uk) in order to identify additional potentially eligible guidelines. Finally, we also searched PubMed with the strategy (guidelines OR recommendations OR position statement) AND (breast OR mammary OR ovarian OR ovary OR endometrial OR endometrium OR uterine OR cervical OR cervix) AND (cancer OR carcinoma OR neoplasm OR neoplasia).

### Data extraction – eligible websites

For each pertinent society or organization with an accessible website, we recorded its name, URL, continent and/or country(ies), specialty setting (medical oncology, radiation oncology, surgical oncology, cancer research, or gynecology), and whether it provided any clinical practice guidelines on any topic, regardless of whether the guidelines had been developed by the specific society/organization or some other source and similarly whether they provided any eligible guideline addressing chemotherapy for one or more of the eligible malignancies. Whenever any eligible guidelines were available, we recorded whether recommendations were freely accessible through the website and whether they provided separate information developed by the society/organization itself or a link to another society/organization's guidelines.

### Data extraction – eligible guidelines

We recorded the exact wording and documentation regarding the chemotherapy question from all the eligible guidelines that mentioned anything on chemotherapy of advanced or recurrent breast, ovarian, cervical and endometrial cancer. For cervical cancer we separately recorded statements for stages III–IVa and stage IVb since treatment and chemotherapy response differs between these stages [13], [16], [17].

For each eligible guideline we addressed whether it provided grading for the levels of evidence (and how this had been assessed) and the strength of recommendations. We recorded aspects of the production and implementation process for the guideline, including involvement of multidisciplinary teams, search strategies, reported funding, reported conflicts of interest, implementation plans, and use of specific indicators to assess actual uptake. In addition, we recorded the date of publication of the guidelines and the date of publication of the most recent cited randomized trial or meta-analysis. We addressed the utilization of randomized evidence within the guidelines by recording whether guidelines cited within their reference list any randomized trials and meta-analyses pertinent to the chemotherapy question and, if so, how many; and whether they made a comment on the need for more randomized trials to provide sufficient guidance.

We also analyzed the outcomes discussed in the guidelines. We addressed whether the guidelines provided any statement regarding each of the following aspects of the effects of chemotherapy: overall survival, disease-free interval or progression-free survival, response rate, recurrence rate, symptoms relief, quality of life, toxicity profile and cost.

Finally, for each guideline and each type of advanced stage malignancy, we recorded the position on whether chemotherapy should be used or not. We classified guidelines as “in favor” or “against”, if they clearly supported or clearly opposed, respectively, the use of chemotherapy. We also recorded whether guidelines in favor of chemotherapy were unequivocal about its overall favorable benefit-risk ratio or offered any caveats.

### Analyses

We evaluated whether guidelines for the different malignancies differed among themselves in the use of grading, aspects of the production and implementation process, timeliness, utilization of randomized evidence, outcomes discussed, and stance towards use of chemotherapy. Group comparisons for categorical variables used Fisher's exact test and group comparisons for continuous variables used Mann-Whitney U test and analysis of variance. All analyses were conducted in STATA SE version 10.0. P-values are two-tailed.

## Results

### Eligible societies and organizations

Internet searches identified 220 societies/organizations. Additional searches performed in Pubmed and standard guideline websites identified 4 more societies (1 and 3 respectively) that provided any sort of clinical practice guidelines. Of the 224 entities, 105 were named “society”, 51 “association”, 8 “organization” and 60 had other names (alliance, board, centers, coalition, college, consortium, council, federation, forum, foundation, fund, group, institute, league, network, school and union). There were 26 international, 77 intercontinental, and 153 national entities covering a diverse array of countries and a range of specialties (Table 1). Nine of the 224 did not have an accessible webpage, 23 had no functional webpage at all, and 6 had restricted access, thus 192 entities could be accessed and evaluated for the presence of guidelines. Most of them (n = 149) had a webpage in English (Table 1).

**Table 1:** Distribution of organizations by location, society and organization type.

	Eligible (accessible) societies and organizations	Number with a website in English	Number with guidelines
***Continent***			
International	26 (22)	22	6
America(a)	35 (33)	32	16
Europe	16 (16)	16	8
Africa	4 (3)	3	0
Asia	7 (5)	5	1
Australia & New Zealand	15 (14)	14	9
***Country***			
USA (a)	32 (31)	31	16
Canada	13 (11)	11	3
North Europe			
Sweden	3 (1)	1	1
Norway	6 (4)	2	1
Iceland	3 (2)	1	0
Finland	3 (1)	1	0
Denmark	5 (3)	3	0
Ireland	6 (3)	3	0
UK	11 (11)	11	5
Central & Western Europe			
Austria	6 (1)	1	2
Spain	7 (7)	3	1
Switzerland	10 (9)	4	2
France	13 (12)	2	4
Netherlands	8 (6)	1	2
Belgium	8 (8)	5	2
Luxemburg	1 (1)	0	1
Italy	9 (9)	3	5
Japan	9 (5)	5	0
***Society type***			
Gynaecology	64 (53)	35	21
Overall cancer	53 (47)	38	17
Medical Oncology	22 (13)	10	4
Radiation oncology	23 (20)	15	6
Surgical Oncology	11 (10)	6	3
Cancer research	24 (23)	21	7
Other (b)	27 (26)	24	11
***Total***	**224 (192)**	**149**	**69**

(a) Continental American organizations include all USA national organizations along with 2 other South American organizations.

(b) “Other” refers to any society setting that does not belong to any of the above specialty settings. This may include other specialty societies or other societies such as guideline developers.

### Availability of any guidelines

Sixty-nine entities (31%) provided any sort of guidelines in their websites (Table 1). For fifty-nine of them, at least one guideline had been developed by the society/organization itself and in the other 10 there were only links to other societies/organizations' guidelines. Fifty two (75%) made their guidelines available in the English language. The other 17 (25%) provided only guidelines in other languages (4 Italian, 3 German, 3 French, 1 French and German, 2 Dutch, 1 Norwegian, 1 Belgian and 1 Luxemburg). Availability of guidelines did not vary per geographic location and specialty setting (Table 1).

### Eligible guidelines for chemotherapy in advanced gynecological malignancies

Twenty entities provided any sort of guideline for chemotherapy in advanced or recurrent gynecological cancer (Table 2). Among them, 14 had developed at least one eligible guideline in English language on their own. This included 8 entities with eligible guidelines for breast cancer chemotherapy [18]–[25], 10 for ovarian cancer [18]–[27], 8 for cervical cancer [18]–[22], [25]–[27], and 8 for endometrial cancer [18]–[22], [26]–[28]. Five had developed guidelines for chemotherapy for all these malignancies [18]–[22].

**Table 2:** Entities with guidelines for advanced stage gynecologic malignancies.

Entity	Link	Breast cancer	Ovarian cancer	Cervical cancer	Endometrial cancer
American Cancer Society	www.cancer.org	Own-developed guideline	Own-developed guideline	Own-developed guideline	Own-developed guideline
American College of Obstetricians and Gynecologists	www.acog.org	No guideline	No guideline	No guideline	Own-developed guideline
American Society for Therapeutic Radiology and Oncology	www.astro.org	Link to other	Link to other	Link to other	Link to other
Association of Residents in Radiation Oncology	www.arro.org	Link to other	Link to other	Link to other	Link to other
Australian Gynecological Cancer Society	www.gcsau.org	No guideline	Own-developed guideline	Own-developed guideline	Own-developed guideline
British Association of Cancer United Patients	www.cancerbackup.org.uk	Link to other	Link to other	Link to other	Link to other
Canadian Association of General Practitioners in Oncology	www.cos.ca/cagpo	Link to other	Link to other	Link to other	Link to other
Cancer Council Australia	www.cancer.org.au	No guideline	Own-developed guideline	No guideline	No guideline
European Society for Medical Oncology	www.esmo.org	Own-developed guideline	Own-developed guideline	Own-developed guideline	Own-developed guideline
International Federation of Gynecology and Obstetrics	www.figo.org	No guideline	Own-developed guideline	Own-developed guideline	Own-developed guideline
International Gynecologic Cancer Society	www.igcs.org	No guideline	Own-developed guideline	Own-developed guideline	Own-developed guideline
Medical Oncology Group of Australia	www.moga.org.au	Link to other	Link to other	Link to other	Link to other
National Cancer Institute	www.cancer.gov	Own-developed guideline	Own-developed guideline	Own-developed guideline	Own-developed guideline
National Comprehensive Cancer Network	www.nccn.org	Own-developed guideline	Own-developed guideline	Own-developed guideline	Own-developed guideline
National Foundation for Cancer Research	www.nfcr.org	Own-developed guideline	Own-developed guideline	Own-developed guideline	Own-developed guideline
National Health and Medical Research Council	www.nhmrc.gov.au	Own-developed guideline	Own-developed guideline	No guideline	No guideline
National Institute of Health and Excellence	www.nice.org.uk	Own-developed guideline	Own-developed guideline	No guideline	No guideline
Royal College of Obstetricians and Gynaecologists	www.rcog.org.uk	Link to other	No guideline	No guideline	No guideline
Scottish Intercollegiate Guidelines Net work	www.sign.ac.uk	Own-developed guideline	Own-developed guideline	Own-developed guideline	No guideline
Society of Gynecologic Oncologists	www.sgo.org	No guideline	No guideline	No guideline	Own-developed guideline

### Grading and timeliness

A little over half of the guidelines covering the use of chemotherapy in advanced gynecological malignancies addressed the level of evidence available for the recommendations; nonetheless no uniformity existed regarding the systems for grading the level of evidence with 5 different systems being utilized (Table 3). Furthermore, less than half provided a grading system for the recommendations with no significant differences across the examined malignancies (Table 3). The timeliness of the guidelines was also comparable among the malignancies tested (Table 3). About two-thirds of the guidelines had been published within the last 5 years and more than half within the last 2 years. No significant difference was observed across guidelines for the different malignancies neither for the date of publication of the most recent cited trial or meta-analysis nor for the number of cited trials or meta-analyses published within the last 5 years.

**Table 3:** Methods of development and utilization or randomized evidence in guidelines for advanced gynecological malignancies.

	Type of cancer					P-value
	Breast cancer	Ovarian cancer	Cervical cancer III–IVa	Cervical cancer IVb	Endometrial cancer	
**Number of available guidelines**	8	10	8	8	8	
**Grading system of evidence and recommendations**						
Level of evidence*	5 (63%)	5 (50%)	4 (50%)	5 (63%)	4 (50%)	0.96
NCI PDQ ranking system (1)	1	1	1	1	1	
SIGN grading system (2)	2	1	1	1	0	
Grading system used by ASCO (3)	1	1	1	1	1	
NHMRC grading system (4)	1	1	0	0	0	
US Preventive Services task force (5)	0	0	0	1	1	
Not described (6)	0	1	1	1	1	
Grade of recommendation	3 (38%)	3 (30%)	3 (38%)	3 (38%)	2 (25%)	1.00
**Key aspects for guidelines production process**						
Guidelines' panels						0.88
Multidisciplinary	6 (75%)	7 (70%)	5 (62%)	5 (62%)	5 (62%)	
Only one discipline	0	0	0	0	1 (13%)	
Unclear	2 (25%)	3 (30%)	3 (38%)	3 (38%)	2 (25%)	
Description of the search strategy used						1.00
Yes	3 (38%)	3 (30%)	2 (25%)	2 (25%)	2 (25%)	
No	5 (62%)	7 (70%)	6 (75%)	6 (75%)	6 (75%)	
Funding						0.95
Non-industry or no funding	6 (75%)	6 (60%)	5 (62%)	5 (62%)	4 (50%)	
Not reported	2 (25%)	4 (40%)	3 (38%)	3 (38%)	4 (50%)	
Reporting of members' conflicts of interest						0.95
Yes	4 (50%)	4 (40%)	3 (38%)	3 (38%)	2 (25%)	
No	4 (50%)	6 (60%)	5 (62%)	5 (62%)	6 (75%)	
Implementation plan described						0.64
Yes	4 (50%)	4 (40%)	2 (25%)	2 (25%)	1 (13%)	
No	4 (50%)	6 (60%)	6 (75%)	6 (75%)	7 (77%)	
Performance indicators to assess guidelines uptake						0.64
Yes	4 (50%)	4 (40%)	2 (25%)	2 (25%)	1 (13%)	
No	4 (50%)	6 (60%)	6 (75%)	6 (75%)	7 (77%)	
**Timelines of guidelines updates**						
Guideline publication date (median and range)	2008(2001–2009)	2007(2001–2009)	2008(2001–2009)	2008(2001–2009)	2008(2001–2009)	0.97
Guidelines published within the last 5 years	6 (75%)	6 (60%)	7 (88%)	7 (88%)	6 (75%)	0.66
Guidelines published within the last 2 years	5 (63%)	5 (50%)	5 (63%)	5 (63%)	4 (50%)	0.97
Date of the most recent cited randomized trial or meta-analysis (median and range)	2008(2000–2008)	2006(1996–2007)	2005(1999–2007)	2005(1985–2007)	2006(2004–2006)	0.22
Cited randomized trial or meta-analysis published within the last 5 years	6 (75%)	5 (50%)	3 (38%)	3 (38%)	4 (50%)	0.81
**Utilization of randomized evidence**						
Citation of any randomized trial	7 (88%)	9 (90%)	3 (38%)	4 (50%)	4 (50%)	0.09
Number of cited randomized trials (mean)	11.88	9.9	1.63	1.25	1.38	<0.001
Citation of any meta-analysis	6 (75%)	3 (30%)	2 (25%)	1 (13%)	0	0.013
Number of meta-analyses (mean)	1.38	1.1	0.63	0.13	0	0.19
Need for more randomized trials	6 (75%)	7 (70%)	3 (38%)	5 (63%)	4 (50%)	0.62

*the reference sources for the assignment of levels of evidence are as follows:

(1) http://www.cancer.gov/cancertopics/pdq/levels-evidence-adult-treatment/HealthProfessional/page2;

(2) Scottish Intercollegiate Guidelines Network. Methodology Review Group. Report on the review of the method of grading guideline recommendations. Edinburgh: SIGN; 1999.;

(3) Cook DL, Guyatt GH, Laupacis A, et al: Rules of evidence and clinical recommendations on the use of antithrombotic agents. Chest 102:305S–311S, 1992 (suppl 4);

(4) National Health and Medical Research Council (NHMRC). How to Use the Evidence: Assessment and Application of Scientific Evidence. Canberra, Australia: NHMRC; 2000;

(5) http://www.uspreventiveservicestaskforce.org/uspstf08/methods/procmanual4.htm;

(6) FIGO guidelines present a grading system for level of evidence (A–D) without specifying which system used.

### Key aspects of the production and implementation process

Key aspects regarding the guideline production and implementation process did not significantly differ among analyzed guidelines. More than 60% of the guidelines were stated to have been developed by multidisciplinary teams of experts and only 1 guideline regarding endometrial cancer was developed by experts from one medical field only (obstetricians/gynecologists), while in the remaining guidelines it was unclear what experts exactly were involved. A specific search strategy for the development of the guideline was provided in less than 40%. None of the guidelines reported industry-related funding, but often there was no statement about who had potentially funded the guidelines; moreover, conflicts of interest statements from panel members were available in less than half of the guidelines. Finally, only few of the guidelines provided an implementation plan of the guidelines beforehand or reported that specific indicators were considered in order to assess their actual uptake (Table 3).

### Utilization of randomized evidence

The number of randomized trials cited within the guidelines significantly differed among the malignancies tested (p<0.001) (Table 3). Almost all the guidelines for breast and ovarian cancer cited at least one randomized trial within their statements compared to 50% or less of the guidelines for cervical and endometrial cancer. It was also significantly more likely for meta-analyses to be cited in the former than the latter group of malignancies. However, the number of guidelines suggesting the need for more trials to be conducted was comparable for the malignancies tested.

### Discussion of outcomes

Statements regarding overall survival were provided in most guidelines with no significant difference for the discussion of this outcome for the different malignancies (Table 4). Conversely, discussion of outcomes such as disease-free survival, response rate, symptoms relief, quality of life, and toxicity of medications significantly differed across malignancies with breast and ovarian cancer guidelines providing more frequently such statements compared to cervical and endometrial cancer. In particular, none of the guidelines on stage III–IVa cervical cancer or endometrial cancer touched on issues of costs related to chemotherapy, whereas less than 40% addressed issues regarding toxicity (Table 4).

**Table 4:** Outcomes discussed in guidelines for chemotherapy for advanced gynecological malignancies.

	Type of cancer					P-value
	Breast cancer	Ovarian cancer	Cervical cancer III–IVa	Cervical cancer IVb	Endometrial cancer	
**Number of available guidelines**	8	10	8	8	8	
Overall survival	7 (88%)	8 (80%)	5 (63%)	5 (63%)	5 (63%)	0.68
Disease-free survival	7 (88%)	8 (80%)	2 (25%)	3 (38%)	3 (38%)	0.033
Response rates	7 (88%)	10(100%)	3 (38%)	5 (63%)	5 (63%)	0.024
Recurrence	4 (50%)	6 (60%)	7 (88%)	8(100%)	7 (88%)	0.092
Symptoms relief	4 (50%)	8 (80%)	1 (13%)	6 (75%)	1 (13%)	0.005
Quality of life	6 (75%)	5 (50%)	0	5 (63%)	0	0.001
Toxicity	7 (88%)	8 (80%)	3 (38%)	2 (25%)	3 (38%)	0.039
Costs	3 (38%)	2 (20%)	0	0	0	0.054

### Stance towards use of chemotherapy

None of the analyzed guidelines for these advanced stage gynecological malignancies were against using chemotherapy in these patients. All guidelines seemed to endorse explicitly or implicitly the use of some chemotherapy, at least in some settings and patients. No major caveats were raised for breast, ovarian, and stage III–IVa cervical cancer [18]–[27]. For stage IVb cervical cancer three guidelines had some caveats stating that treatment should be individualized (“Patients with stage 4 disease have their treatment very much individualized depending on the distribution of the disease” [27]) or that no standard chemotherapy is available (“No standard chemotherapy treatment is available for patients with stage IVB cervical cancer that provides substantial palliation” [21] and “There is no good single chemotherapy approach that can improve the length of survival in patients with metastatic cervical cancer. Unfortunately, these chemotherapies typically work for only a few months before the cervical cancer begins to grow again. Most patients ultimately succumb to cancer and better treatment strategies are clearly needed” [22]). For endometrial cancer, 3 guidelines only presented data from trials without clear supportive statements that patients would benefit from chemotherapy [20], [21], [27]. All guidelines, with the exception of 1 on endometrial cancer and 1 on cervical cancer, also presented information on and/or endorsement of specific chemotherapeutic agents.

## Discussion

Our evaluation identified over 200 cancer and gynecological societies and organizations that operate at international or national level. This is an impressive number and it offers a picture of flourishing professional activity. Many of these organizations have very extensive membership, organize large meetings, and have substantial influence upon their members, subscribers, and visitors to their websites [29]–[32]. Yet only a third of these societies/organizations provided any sort of guidelines within their websites. When it comes to specific questions of interest, such as the use of chemotherapy in advanced stage gynecological malignancies that we analyzed here, only one in ten societies/organizations offered such guidelines, and an even smaller number of them had developed their own guidelines. Therefore, a few professional entities seem to dominate the literature of these influential documents; some others adopt what is produced by others, while the majority is unfamiliar with the guideline concept. Important aspects of the production and implementation process of these guidelines were often unstated. The number of available guidelines did not depend on the bulk of available evidence or the strength of the evidence in favor of chemotherapy. A similar number was available for advanced breast and ovarian cancer where hundreds of trials have been published [11], [12] as well as for advanced endometrial and cervical cancer where the evidence is more limited [13], [14].

The analyzed guidelines did not differ across malignancies in terms of their adoption of levels of evidence, grading of recommendations, and aspects of production and implementation process, or timeliness. Their performance in this regard on average is comparable to guidelines from other fields [33]–[40] and there is substantial room for improvement. The use of levels of evidence and grades of recommendation may be enhanced if this approach is adopted centrally for all guidelines produced by an organization, e.g. the ACC/AHA guidelines in cardiology always use this approach [9]. Timeliness of guidelines updates also constitutes a serious issue [8] and it can affect the reliability of these documents, especially for fields where evidence changes rapidly. There is large variability across medical fields and across clinical questions of interest in the rate with which new evidence accumulates and changes the overall picture [41]–[44]. For all the malignancies analyzed here, most guidelines were updated within the last 5 years. This is probably an acceptable frame for these fields, although it is still possible that a window of 5 years may miss some important recent randomized evidence on specific regimens. One may need to re-assess the acceptable time frame as new evidence arises, but most guidelines developers lack formal procedures for updating their guidelines [45].

The guidelines that we analyzed differed substantially across malignancies in the extent of utilization of randomized evidence (citation of randomized trials and meta-analyses). This is not surprising, given that there are far more randomized trials and meta-analyses performed for advanced breast and ovarian cancer than for cervical and endometrial cancer. The conduct of randomized trials may be encouraged in fields where results of chemotherapy have been favorable (breast and ovarian cancer), while investigators may feel less enthusiastic to perform more trials in fields where chemotherapy has shown equivocal results with no clear benefits (cervical and endometrial cancer). Most of the analyzed guidelines cited at least one randomized trial. It has been observed across diverse fields that utilization of randomized evidence has increased in guidelines over time [46]. Nevertheless, most of the analyzed guidelines cited few randomized trials of those performed on the respective questions. Therefore use of randomized evidence seems to remain eclectic in these documents.

Even though the benefits of chemotherapy vary a lot among the malignancies that we analyzed, all of the guidelines explicitly or implicitly endorsed the use of chemotherapy for all malignancies. This is fully justified for breast, ovarian and possibly stage III–IVa cervical cancer, where the available evidence shows that chemotherapy has substantial benefits [11], [12], [16], [17]. Conversely, chemotherapy has shown no convincing benefits, especially for hard outcomes such as survival, for stage IVb cervical and advanced endometrial cancer [13], [14]. However, only 3 of the 8 guidelines for each of the latter types of tumors had some caveats and were not openly committed to widespread use of chemotherapy in these patients.

Furthermore, outcomes such as secondary effectiveness measures and toxicity profile were addressed more frequently in breast and ovarian cancer, whereas cervical and endometrial cancer guidelines totally omitted statements regarding quality of life and costs of chemotherapy and were less likely to discuss also these other outcomes. Reporting of these issues is important for one to adopt or decline the use of specific treatments. Especially in tumors in which chemotherapy does not appear to offer clear benefit in terms of overall survival, secondary effectiveness outcomes, toxicity, quality of life and cost may be the only data available to justify or not its use. Omission of statements regarding these outcomes in these malignancies may mislead conclusions and may explain the generally favorable stance of the guidelines towards chemotherapy even for advanced cervical and endometrial cancer.

One may argue that scientific societies may sometimes have considerable conflicts that pose obstacles in creating objective guidelines. For example, if professionals benefit from administering specific interventions to patients, their societies may not be unbiased enough to recommend that patients should not be treated with specific interventions (such a recommendation would reduce the volume of clientele and financial gains of these professionals). The ubiquitous recommendation of chemotherapy for all the examined types of cancers (regardless of whether it is effective or not) raises such concerns. An unbiased, intersocietal and interprofessional collaborative approach may be useful in this regard.

Some limitations of our work should be discussed. First, for 32 entities we could not find access to a website. However, it is not very likely that these entities would have guidelines of their own, let alone high-quality ones. Second, it is possible that some entities may not have published guidelines on chemotherapy, if they deemed that chemotherapy is ineffective and thus not worth addressing. However, we found a similar number of guidelines for tumors where chemotherapy is very effective and for those where it is more controversial. Third, there are no established validated searches for unearthing professional societies and organizations and some of them may have been missed by our searches. However, given the multiple layers of our search, it is unlikely that prominent entities were missed.

In conclusion, our evaluation suggests that guidelines from professional gynecological and cancer societies and organizations have substantial room for improvement. Recommendations should be based on solid scientific evidence with balanced discussion of all potential benefits, harms and costs of the proposed interventions.
